# Medical diversity of migrants’ physical and mental health understandings

**DOI:** 10.1016/j.jmh.2026.100438

**Published:** 2026-08-22

**Authors:** Kevin Becker, Frauke Kraas, Jonas Birke, Carsten Butsch

**Affiliations:** a Department of Geography, University of Bonn, Mseckenheimer Allee 166, 53115, Bonn, Germany; b Department of Geography, University of Cologne, Otto-Fischer-Straße 4, 50674, Köln, Germany

**Keywords:** Migration, Health, Understandings of health, Medical diversity, Germany, Urban health

## Abstract

Health understandings of migrants, immigrants and persons with a migration background (in short: 'im/migrants') often diverge from the biomedical paradigm dominant in many host societies. Shaped by culturally diverse backgrounds, these understandings influence im/migrants’ health practices and utilisation of formal healthcare services.

This study aims to explore the health understandings of im/migrants residing in two socio-culturally diverse urban neighbourhoods: Bonn-Tannenbusch and Cologne-Mülheim, Germany. Employing a practice-theory approach (Schatzki 1996, 2002), a cross-sectional survey was conducted using a standardised online questionnaire. The instrument was developed based on earlier qualitative research findings. In total, 179 im/migrants and 172 respondents without an im/migration background participated in this study.

Based on the collected data, two typologies of practice-shaping understandings of physical and mental health were developed. Several types of understandings were identified, each comprising varying combinations of explanatory concepts, which can be grouped into three broad categories: biomedical, alternative medical, and supernatural. A more detailed analysis was conducted on the identified types using descriptive statistics. This analysis showcases how health understandings differ from each other and how, in some cases, they share certain similarities.

The findings underline that medical diversity, characterised as the coexistence and sometimes blending of ontologically diverse understandings and practices, is not marginal, but rather a pervasive feature of im/migrants’ health understandings and practices in the two study sites. This article contributes to the knowledge on the diversity of health understandings in diverse neighbourhoods, and provides insight into the factors that influence the adoption or rejection of specific health practices.

## Highlights

• Respondents with a migration background explain health with biomedical, alternative and supernatural concepts.
• Respondents construct their understandings by borrowing from different concepts.
• Respondents’ understandings of physical and mental health can differ strongly.
• Refinement of a typology of health and disease understandings.
• Medical diversity is shaped by diverse cultural backgrounds.

## 1 Introduction

Health understandings are inter alia influenced by cultural and educational background (Kleinman, 1980; Michlig et al., 2022). They are negotiated in peer groups, including both local and translocal networks – in the host society, the society of origin, or in "diaspora" contexts. Additionally various local and translocal institutions (e.g., educational, health and religious institutions) shape health understandings (Becker et al., 2026; Kleinman, 1980; Okoror et al., 2025; van der Meer et al., 2023; Yang et al., 2014). Following Schatzki's (1996, 2002) practice-theory perspective, health understandings can be formed as “general understandings” (2002, 77), affecting an individual’s health practices (Becker et al., 2026). Globalisation, and notably international migration, have resulted in the exchange of different understandings of health, which subsequently give rise to an array of health practices (Dilger and Schnepf, 2020; Krause, 2008; Krause et al., 2012). Consequently, individuals, especially im/migrants, who hold specific formative concepts for health, may encounter novel concepts following im/migration or through exchange with new peers at the place of residence. These may initially be accepted only, thus having little influence on the individual explanation for health, but later become formative concepts, subsequently forming new understandings resulting in the emergence of new health practices.

In host countries, such as Germany, where over 30% of the population have an im/migration background (DESTATIS, 2025), it seems essential to research the growing diversity of health understandings and practices. This will allow healthcare providers to develop more culturally sensitive health services and thus improve access for this diverse population group.

Against this backdrop, the project 'Migrant understandings of health and their health-related practices in diverse neighbourhoods' (MiGeQua) aims to explore and advance the theoretical development of culturally informed understandings of health. The primary objective of the present study was to examine the prevalence of diverse practice-shaping health understandings of im/migrants in two urban neighbourhoods, Bonn-Tannenbusch and Cologne-Mülheim, and to develop robust typologies of im/migrants' understandings of physical and mental health.

Drawing upon practice-theory (Schatzki, 1996, 2002), the cross-sectional survey presented here allows for: (1) the development of two comprehensive typologies of im/migrants' physical and mental health understandings; (2) in depth investigation of im/migrants' "medical diversity" (Parkin, 2013, 125); and (3) an analysis of diverging understandings of physical and mental health.

The remainder of this article is structured as follows. The second section outlines the theoretical background, followed by a detailed description of the methodology, including study design, sampling and recruitment, questionnaire development, analytical procedures, and study sites. The results section then presents participants’ sociodemographic characteristics, the developed typologies of im/migrants’ understandings of physical and mental health, and an assessment of medical diversity. These findings are discussed in relation to existing literature. The article concludes with a summary of the study’s strengths and limitations, and the key takeaways.

## 2 Theoretical background

This section discusses explanatory concepts of health identified in prior research on im/migrants and introduces the analytical concept of “medical diversity” (Parkin, 2013, 125). It then outlines existing typologies of health understandings and briefly presents Schatzki’s practice theory (1996, 2002), which informs the conceptualisation and empirical analysis of health understandings and practices in this study.

Im/migrants’ health understandings and practices are influenced by several biomedical, alternative medical and supernatural explanatory concepts, which may diverge from those dominant in host societies. Earlier studies investigated this, e.g., for Germany (Becker et al., 2026; Grupp et al., 2018; Samerski, 2019; Shu et al., 2022) and for other countries, such as England (Green et al., 2006), Denmark (Ramzan et al., 2016) and the United States (Crocker et al., 2024; Jiang and Quave, 2013). *Biomedical explanatory concepts* focus on measurable disorders, which are explained by the complex interaction in various domains like biological, behavioural, and environmental factors. For instance, they include lifestyle and behavioural factors, such as diet and exercise (van Apeldoorn et al., 2025; van der Meer et al., 2023; Yilmaz-Aslan et al., 2014), and biological processes, such as ageing and genetic predispositions as health determinants (Shu et al., 2022; van der Meer et al., 2023; Yilmaz-Aslan et al., 2014). *Alternative medical explanatory concepts* include notions about the human body, where health and disease are linked to bodily purity or harmful bodily fluids, or the idea of (im-)balance, where health is seen as a state of equilibrium and disease results from bodily, mental, or environmental imbalance (Dilger and Schnepf, 2020; Kotte, 2010). *Supernatural explanatory concepts* are divided into beliefs about deities, where health and disease were perceived as consequences of divine intervention (Franz et al., 2007; Grupp et al., 2018; van Apeldoorn et al., 2025; van der Meer et al., 2023; Yilmaz-Aslan et al., 2014), and beliefs about supernatural processes, which include beliefs in shamanism, spirits, and fate as health determinants (Grupp et al., 2018; van der Meer et al., 2023; Yilmaz-Aslan et al., 2014).

Medical anthropology has in the past engaged in the study of varied cultural interpretations of health, disease, and associated practices in diverse social contexts (Gauliver, 2024; Omobowale, 2022). To date, a number of studies have categorised the different understandings and explanatory concepts for physical and mental health. In general, these studies either (1) categorised solely understandings for mental health (Eisenbruch, 1990), (2) placed emphasis on categorising the understandings held by individuals from specific origins (Foster, 1976), or (3) sought to categorise either largely biomedical or non-biomedical health understandings (Erickson, 2008; Faltermaier et al., 1998).

Research addressing simultaneously physical and mental health understandings of im/migrants remains limited. Exceptions include the work of van der Meer et al. (2023) and Lujic (2008). Van der Meer et al. (2023) show that young refugees in Germany explain physical health, for instance, through lifestyle-related factors such as smoking, alcohol consumption, insufficient sleep, and adverse weather conditions, while mental health is attributed to traumatic experiences, stress, and separation from family members. Lujic’s (2008) study illustrates that Turkish im/migrants explain physical disease, for instance, through environmental and structural factors or divine intervention, whereas mental illness is linked to supernatural causes, insufficient willpower, stress, or familial conflict.

As im/migrants frequently blend multiple explanatory concepts, they create individualised health understandings (Becker et al., 2026). The emergence of such blended health understandings and the simultaneous use of diverse health practices, has been conceptualised as medical pluralism (Gilbert et al., 2019; Green et al., 2006; Main, 2016). In a conceptual advancement, Parkin (2013), 125 highlights, pluralism usually denotes to the relatively isolated coexistence of medical traditions and suggests "medical diversity" (2013, 125) as an alternative, which emphasises mutual borrowing of ideas, practices and styles. It thus, better captures the mixtures and adaptations of health understandings and practices identified for our sample (Krause et al., 2012; Parkin, 2013; cf. also Section 3.3).

Schatzki’s practice-theory (1996, 2002) helps to consistently frame the nexus between im/migrants’ health understandings and practices. It provides from our perspective a consistent explanatory framework for understanding health practices, yet its application in this domain has been rare. One exception is Samerski’s (2019) research, who examined the health literacy of im/migrants residing in diverse urban neighbourhoods, identifying a range of concurrent health understandings of participants. The theory conceptualises practices as consisting of "doings" and "sayings", structured by practical and general understandings, rules, and teleoaffective structures (Schatzki, 2002, 77). In the present work, *health understanding* is predicated on Schatzki's concept of "general understandings" (2002, 77). Following Schatzki, practices occur within a "space of places" (1996, 115), shaped by interactions that intertwine individuals' lives. Health understandings may evolve through encounters between individuals with diverse im/migration backgrounds, leading to modifications in practices. These adaptations can generate new health understandings and consequently transform the spatial and social contexts in which health practices are performed. Thus, im/migration fosters dynamic evolution of health understandings, health practices and their spatial and social contexts.

## 3 Methods, materials and study sites

### 3.1 Study design

This study is part of the research project MiGeQua for which an integrated mixed-methods research (MMR) design (Axinn and Pearce, 2006; Schäfer et al., 2018; Texier-Ast, 2018) has been developed. In this paper we present findings from a cross-sectional survey conducted from July 2024 to February 2025. This method was employed to explore the diverse understandings of physical and mental health held by im/migrants residing in Bonn-Tannenbusch and Cologne-Mülheim. As part of the MMR-design, this study aimed at (1) testing a typology developed with qualitative research methods in an earlier research phase (Becker et al., 2026), (2) quantifying the prevalence of broader health understandings, and (3) identifying additional data-driven understandings of physical and mental health.

### 3.2 Inclusion criteria and sampling design

The survey was open to all residents of Cologne-Mülheim and Bonn-Tannenbusch irrespective of their sex, gender or ethnicity, who provided written consent. Individuals under the age of 18 were not included in the survey. The target sample size was 350 participants, with 175 from each neighbourhood. The sample size was calculated using OpenEpi (OpenEpi, 2026). The recommended sample size for a population of 60,000 individuals, with an estimated 50% population with a migration background, and an objective of a confidence interval of 80% is 164. For a confidence interval of 95%, the suggested sample size is 382. In light of the challenges encountered in engaging these “hard-to-reach groups” (Bekteshi et al., 2024), a sample size of 350 was deemed feasible from a logistic point of view. To ensure the adequate inclusion of im/migrants, as recommended by Xi et al. (2022), we employed a combination of probability and non-probability sampling methods.

#### 3.2.1 Probability sampling

First, a random sample was drawn using the QGIS. All attributes designated as 'building' by OpenStreetMap within the statistical districts of both neighbourhoods were retrieved utilising the QGIS plugin QuickOSM. Buildings in which no residential units were conceivable (e.g., churches, schools etc.) were excluded from the further procedure.

To achieve the desired sample size of 150 participants per neighbourhood, it was decided to include 66% of all residential complexes in Bonn-Tannenbusch (n = 1.366) and 26% in Cologne-Mülheim (n = 1.390) in the recruitment process. A digital randomisation method was employed, coded in QGIS. For each building a random value between 1 and 100 was automatically assigned; only those buildings with numbers 1 to 66 in Bonn-Tannenbusch and 1 to 26 in Cologne-Mülheim were included in the recruitment process.

#### 3.2.2 Recruitment of respondents

Data collection started on 16th of July 2024 in Bonn-Tannenbusch and on 19th of August 2024 in Cologne-Mülheim and was conducted by the first author in collaboration with one male and one female research assistant, as it was anticipated that female respondents would be more inclined to engage in conversations with a female and vice versa.

Given the high number of buildings (n = 2756), which in most cases had several residential units, the recruitment strategy was to contact half of the households through a face-to-face invitation at their doorstep. In cases where potential participants were not available, prepared flyers (supplementary files 1 and 2) were left in their letterbox inviting members of the households to take part in the study. The remaining half of the potential households were invited solely by leaving one of the aforementioned flyers in the mailbox. This procedure resulted in 6482 flyers in Bonn-Tannenbusch and 9628 flyers in Cologne-Mülheim being either personally handed over to potential participants or left in letterboxes. As a result, in Bonn-Tannenbusch 149 individuals and in Cologne-Mülheim 147 individuals completed the questionnaire.

#### 3.2.3 Step 3: Facility-based sampling

Preliminary analyses of the socio-demographic data of these 296 respondents revealed that residents with an im/migration background were underrepresented in the sample when compared to the share of the im/migrant population according to official statistics (City of Bonn, 2026a, 2026b, 2026c, 2026d; City of Cologne, 2026a, 2026b). This outcome was anticipated, as im/migrants are frequently categorised as a "hard-to-reach group" or "hidden population" (Blukacz et al., 2023; Font and Méndez, 2013). In view of the difficulties encountered in accessing im/migrants during the first data collection phase, it was decided to undertake in a second step a non-probabilistic sampling as a supplement to increase the share of this group, as suggested by Xi et al. (2022).

This supplement was collected with a facility-based sampling from 7 January 2025 onwards in Bonn-Tannenbusch and from 8 January 2025 onwards in Cologne-Mülheim. The first author introduced the questionnaire to potential participants at events organised by various migrant organisations in both neighbourhoods. Furthermore, a total of 550 flyers were distributed in the offices of migrant organisations and participation in the study was advertised on the multilingual social media channels of these organisations. This strategy proved effective: an additional 45 individuals in Bonn-Tannenbusch participated in the survey. Of these, 31 completed the questionnaire, 30 of whom had an im/migration background. In Cologne-Mülheim, an additional 38 persons participated, 25 completed the questionnaire, 24 of whom had an im/migration background. Data collection was concluded on 14 February 2025. The total number of participants was 604, distributed as follows: 302 in Bonn-Tannenbusch and 302 in Cologne-Mülheim. Of these, 351 completed the questionnaire in its entirety and met the established inclusion criteria (179 from Bonn-Tannenbusch and 172 from Cologne-Mülheim).

It is important to acknowledge that facility-based sampling alone often fails to generate reliable samples of target populations (Magnani et al., 2005; Shaghaghi et al., 2011). However, as demonstrated in other studies, the combination of a probability sample (larger share) with a non-probability sample (small supplement) provides statistically reliable results (Xi et al., 2022).

### 3.3 Questionnaire

As to our knowledge no standardized survey tool in the different repositories on survey designs adequately capture the complexity of the diverse and often divergent mental and physical health understandings of im/migrants. For instance, the “Illness Perception Questionnaire Revised” (IPQ-R) (Moss-Morris et al., 2002) only partially reflects the underlying explanatory concepts of health previously recognised for the neighbourhoods im/migrant populations (Becker et al., 2026). Consequently, a new questionnaire was developed, which (1) allowed for a comprehensive investigation of the previously recognised and novel health understandings and health practices of im/migrants, and (2) allowed for quantifying the presence of heterogeneous and diverging health understandings.

The questionnaire is divided into three sections. The first contains questions regarding the understandings and inherent explanatory concepts for physical and mental health of im/migrants. The second section is used to collect socio-demographic parameters, including age, gender, educational qualifications, society of origin, im/migration experience, length of stay in Germany and German language skills. The third section covers the respondents’ preventive and curative health practices.

For the first section, the variables were constructed on the basis of a preceding qualitative study (Becker et al., 2026). In this study, three broad categories of ontologically different explanatory concepts for physical and mental health were identified (cf. Section 2): (1) biomedical, (2) alternative medical, and (3) supernatural explanatory concepts (Becker et al., 2026). Yet, none of these ontologically different explanatory concepts were the only accepted concepts for the interviewed im/migrants. Instead, they combined at least one formative explanatory concept with sometimes one, sometimes several explanatory concepts. This resulted in a complex typology with five (sub)types of health understandings (Becker et al., 2026): Type A, *“supernatural”*, attributes health primarily to supernatural forces and magical influences. Type B, *"blending supernatural and behavioural factors"*, integrates supernatural with interactionist explanatory concepts. Type C, *"(im-)balance"*, is divided into two subtypes: C1, *"spiritual (im-)balance"*, attributes health to spiritual harmony with a deity; and C2, *"alternative medical (im-)balance"* to bodily, mental, and environmental (im-)balance. Type D, *"biomedical"*, attributes health to lifestyle choices, environmental factors, and biological processes.

Once the catalogue of potential survey questions was developed, the questionnaire was transferred to the GDPR-compliant survey tool 'Lime Survey', in both German and English. These two versions were used for pretests with 10 residents of different origins and four experts (e.g., representatives of migrant organisations and neighbourhood management) from both neighbourhoods in order to check and, if necessary, modify and rephrase the questions, ensuring validity and testing whether the wording of the questions would be understandable for the target population. The final questionnaire was translated into seven additional languages: Arabic, Bulgarian, Persian, Polish, Romanian, Russian and Turkish. All translations were carried out by a professional translation agency that employs qualified native-speakers.

The questionnaire comprises a selection of question types, including dichotomous close-ended questions, single- and multiple-choice questions, and questions using a straight six-point Likert scale.

It is made available in the supplementary appendix in English (supplementary file 3).

The internal reliability of the study was then assessed by calculating Cronbach’s alpha and corrected item-total correlations for the theoretically derived multi-item dimensions of the questionnaire. The instrument was designed to capture heterogeneous explanatory concepts rather than a single latent construct. Consequently, reliability was assessed separately for coherent item groups rather than for the full set of items (see Section 4.2 for details).

### 3.4 Data analysis

The analysis focuses on the development of two comprehensive typologies of im/migrants’ physical and mental health understandings. For this analysis, 22 variables were generated with two “anchor questions” in the questionnaire’s first section. The first one pertains to understandings of physical, the second to understandings of mental health.

(1) *You have a disease and suffer from strong headaches. How do you explain the origin of the disease?*
(2) *You have a non-physical disease and suffer from strong inner tension and restlessness. How do you explain the origin of the disease?*

The 22 items operationalised understandings of health through statements representing the aforementioned explanatory concepts for physical and mental health. Participants indicated on a straight six-point bipolar Likert scale (0=absolutely yes, 1=yes, 2=rather yes, 3=rather no, 4=no, 5=absolutely no) the extent to which they agreed with these statements (and the respective explanatory concept).

For the quantitative analyses, all items were recoded so that higher values indicated stronger agreement. Exploratory cluster analyses were conducted to identify broader empirically grounded types of health understandings and the inherent explanatory concepts in the quantitative data. The clustering procedure itself did not rely on predefined cut-off points. However, the subsequent labelling of explanatory concepts as “formative” or “accepted” was based on interpretive thresholds applied to the final k-means cluster centres. Separate analyses were performed for physical and mental health understandings. Initially, hierarchical cluster analyses were conducted utilising Ward’s method and squared Euclidean distances to explore the plausible cluster numbers. Subsequent to this, alternative four-, five- and six-cluster solutions were estimated using k-means clustering, as evidenced by the dendrograms and agglomeration coefficients. The final solutions were selected according to the following criteria: convergence, cluster size, distinctiveness of item profiles, parsimony and substantive interpretability. The resulting clusters were interpreted as broader types of health understandings, which were composed of different explanatory concepts. The present typology is intended to complement, rather than replace the previous qualitative typology (Becker et al., 2026). While the qualitative typology provides analytical depth and highlights specific (at times rare) configurations, the cluster analyses identify the dominant response patterns in the survey data.

To explore whether cluster membership was associated with basic socio-demographic characteristics, bivariate analyses were conducted for age, gender and educational level for both physical and mental health clusters. Chi-square tests with Cramer’s V were used for categorical variables, and Kruskal-Wallis tests were used for age.

### 3.5 Study sites

The two study areas, Bonn-Tannenbusch and Cologne-Mülheim, are characterised by a high degree of im/migration-related diversity, yet they differ considerably in the composition and socio-cultural profiles of their im/migrant populations.

Bonn-Tannenbusch accommodates 17,708 residents originating from 121 different countries (City of Bonn, 2026a, 2026b, 2026c, 2026d). The most relevant countries of origin are Syria (12.54%), Turkey (5.20%), Iraq (4.44%), Morocco (4.10%) and Poland (2.74%) (City of Bonn, 2026a, 2026b, 2026c, 2026d).

Cologne-Mülheim hosts a larger and even more diverse population of 43,182 individuals from 161 nations (City of Cologne, 2026a, 2026b). The Turkish-origin population constitutes the largest group of individuals with an im/migration background (14.45%) and plays a central role in shaping the socio-economic landscape, particularly through the development of a translocal im/migrant economy (City of Cologne, 2026a, 2026b; Kraas, 2005). Other significant communities include those from Bulgaria (4.67%), Poland (2.61%), Ukraine (2.52%), and Italy (2.42%) (City of Cologne, 2026a, 2026b).

## 4 Results

### 4.1 Study participants

Of the 351 participants who completed the questionnaire in its entirety and met the established inclusion criteria, 179 people had an im/migration background. Of these, 99 resided in Bonn-Tannenbusch and 80 in Cologne-Mülheim (Table 1). Detailed sociodemographic information regarding all participants with an im/migration background is presented in Table 2.

**Table 1:** Participant distribution by neighbourhood and im/migration background.

Participant distribution by neighbourhood and im/migration background	Number of participants	% of all participants	% in the respective neighbourhood
**Neighbourhood Bonn-Tannenbusch**			
Participants with an im/migration background	99	28.2	55.3
Participants without an im/migration background	80	22.8	44.7
Total participants (Bonn-Tannenbusch)	179	51.0	100.0
**Neighbourhood Cologne-Mülheim**			
Participants with an im/migration background	80	22.8	46.5
Participants without an im/migration background	92	26.2	53.5
Total participants (Cologne-Mülheim)	172	49.0	100.0
**Both neighbourhoods (total)**			
Participants with an im/migration background	179	51.0	/
Participants without an im/migration background	172	49.0	/
Total participants	351	100.0	/

**Table 2:** Socio-demographic characteristics of participating im/migrants.

Socio-demographic characteristics of participants with an im/migration background in both neighbourhoods (n =179)	Mean	Standard devidation	Number of participants	% of n
**Gender**				
Female			94	52,5
Male			84	46.9
Diverse			1	0,6
**Age**	39,8	14,0	/	/
**Age Groups**				
18–30			55	30.7
31–40			47	26.3
41–55			48	26.8
65–85			29	16.2
**Im/migration background (self-assessment)**				
Turkey			24	13.4
Morocoo			9	5.0
Bulgaria			9	5.0
Afghanistan			8	4.5
Somalia			8	4.5
Syria			8	4.5
Iran			8	4.5
Other			105	58.7
**Educational background**				
No degree			6	3.4
Compulsory schooling (Hochschulreife)			13	7.3
Secondary school degree (Mittlere Reife)			9	5.0
Entrance qualification for universities of applied sciences (Fachhochschulreife)			19	10.6
General and subject-specific entrance qualification for universities (Allgemeine u. fachgebundene Hochschulreife)			39	21.8
University or technical college degree (Abschluss Universität oder Fachhochschule)			72	40.2
Completed vocational training (Abgeschlossene Berufsausbildung)			21	11.7
**Religiosity**				
Religious			79	44.1
Non-religious			100	55.9
**Predominant religious denominations**				
Islam			79	44.1
Christianity			50	27.9
Buddhism			3	1.7
Other			2	1.1
No denomination			45	25.1
**Years lived in the respective neighbourhood**	14,9	12,9	/	/
**Years lived in Germany**	33,5	15,5	/	/

### 4.2 Typologies of im/migrants’ health understandings

In a first step, internal consistency was assessed for the theoretically deducted explanatory concept dimensions. Cronbach’s alpha indicated moderate internal consistency for biomedical explanatory concepts (six items, α = .661), acceptable internal consistency for alternative medical explanatory concepts (three items, α = .736), and very high internal consistency for supernatural explanatory concepts (two items, α = .907). The corrected item-total correlations were satisfactory overall. They exceeded .40 for all biomedical items except “social environment”, exceeded .54 for all alternative medical items, and were .83 for both supernatural items. The item “social environment” was retained due to its theoretical relevance, while the high alpha value for the two-item supernatural dimension should be interpreted with caution.

The study yielded two typologies of im/migrants’ health understandings, a first one regarding physical and a second one regarding mental health, each including five distinct types. These understandings are composed of various explanatory concepts, which reflect different ontological perspectives, and following Schatzki’s practice-theory approach, thereby shape their health practices. One key finding is that respondents regularly hold different understandings for physical and for mental health.

**Fig. 1:**
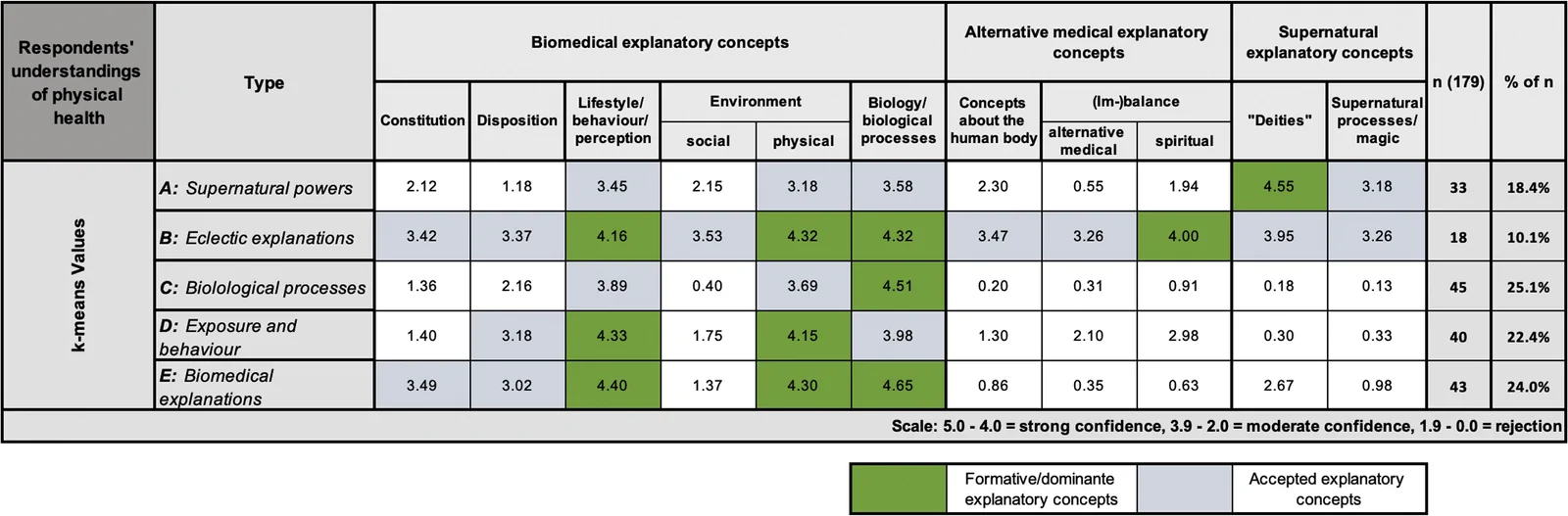
Typology of physical health understandings of respondents.

**Fig. 2:**
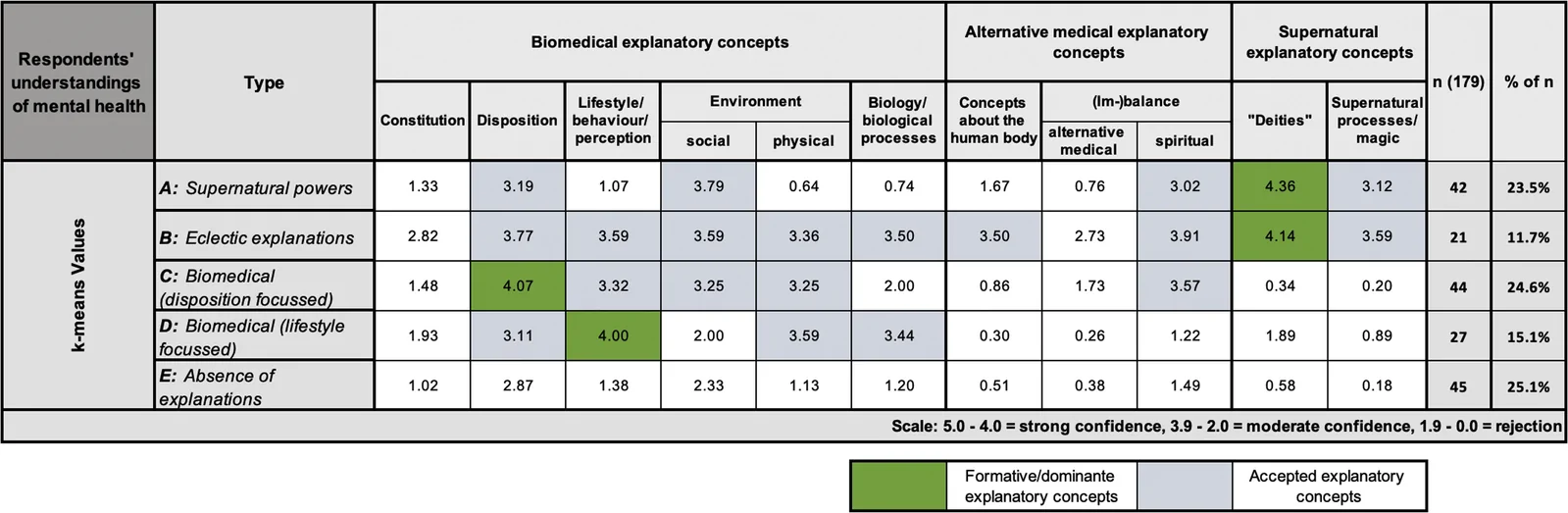
Typology of mental health understandings of respondents.

Both typologies include a variety of "formative explanatory concepts", which serve as the primary determinant of their health understandings (k-means value of ≥4), and/or "accepted explanatory concepts", which exert minor influence on the overall understanding (k-means value of ≥2) (Fig. 1, Fig. 2). It should be noted that the k-means thresholds used to classify inherent explanatory concepts as either “formative” or “accepted” necessarily involve an interpretive cut-off. Consequently, this classification retains an element of qualitative judgement alongside the data-driven clustering procedure.

#### 4.2.1 Im/migrants’ understandings of physical health

##### 4.2.1.1 Physical health understanding A: "supernatural powers"

This cluster consists of thirty-three respondents (18,4%). These individuals exhibited strong confidence in the supernatural explanatory concept, “Deities”. For them the will of divine beings influences human health. Supernatural processes such as magic as well as several biomedical explanatory concepts are additionally accepted explanatory concepts in this cluster (Fig. 1).

This interpretation should be treated cautiously, as the supernatural explanatory dimension was measured with only two items. Although these items showed very high internal consistency, the psychometric basis for interpreting supernatural explanatory concepts is therefore thinner than for the broader biomedical and alternative medical dimensions.

##### 4.2.1.2 Physical health understanding B: "eclectic explanations"

The eighteen im/migrants (10,1%) of this cluster exhibited confidence in explanatory concepts spanning all three major categories (Fig. 1). Formative concepts include biomedical explanatory concepts next to one alternative medical concept, while each of the seven other biomedical, alternative medical and supernatural explanatory concepts were at least accepted. It is this marked diversity of explanatory concepts – or lack of consistency of explanatory concepts – which is distinctive for this cluster. As this cluster also includes supernatural explanatory concepts, its supernatural component should likewise be interpreted cautiously, given that this dimension rests on only two items.

##### 4.2.1.3 Physical health understanding C: "biological processes"

This cluster includes forty-five respondents (25,1%) who strongly endorsed the biomedical explanatory concept of “Biology/biological processes”. They see disease as a malfunctioning of the body, caused by external agents like viruses or processes like ageing. In addition, the respondents expressed moderate confidence regarding other biomedical explanatory concepts, while no confidence was expressed regarding any alternative medical or supernatural explanatory concepts (Fig. 1).

##### 4.2.1.4 Physical health understanding D: "exposure and behaviour"

The forty respondents (22,4%) of this cluster exhibited strong confidence in two biomedical explanatory concepts only: “Lifestyle/behaviour/perception” and “Physical environment”. For them the setting in which life takes place and the individual behaviour exert influence on human health. Additionally, respondents exhibited moderate confidence towards two other biomedical concepts, while expressing no confidence towards alternative medical and supernatural explanatory concepts (Fig. 1).

##### 4.2.1.5 Physical health understanding E: "biomedical explanations"

Forty-three respondents (24,0%) expressed strong confidence in several biomedical explanatory concepts while exhibiting no confidence in alternative medical or supernatural concepts. The participants of this cluster emphasised (1) lifestyle and behavioural choices, (2) the physical environment and (3) biological processes as formative explanatory concepts (Fig. 1).

#### 4.2.2 Im/migrants’ understandings of mental health

##### 4.2.2.1 Mental health understanding A: "supernatural powers"

Forty-two respondents (23,5%) were assigned to this cluster, which is marked by strong confidence solely in the influence of divine beings on mental health, while additionally accepting selected biomedical, alternative medical and supernatural concepts (Fig. 2). The interpretation of supernatural components in mental health clusters should likewise be treated cautiously, as the supernatural dimension rests on only two items despite its very high internal consistency.

##### 4.2.2.2 Mental health understanding B: "eclectic explanations"

The twenty-one respondents (11,7%) of this cluster do not have a coherent health understanding. They borrow from explanatory concepts of all three major categories to assemble an eclectic health understanding, which can be assumed to vary from case to case (Fig. 2). Strong confidence was expressed exclusively towards “Deities”, that is, the influence of divine beings. Furthermore, a degree of confidence was expressed to a certain extent towards all other biomedical, alternative medical and supernatural explanatory concepts, with two exceptions being (1) “Constitution” and (2) “Alternative medical (im-)balance” (Fig. 2).

##### 4.2.2.3 Mental health understanding C: "biomedical (disposition focused)"

The forty-four respondents (24,6%) of this cluster predominantly expressed confidence in biomedical explanatory concepts. Strong confidence was expressed towards the concept “Disposition”, while moderate confidence was expressed toward several other biomedical and “Spiritual (im-)balance” as alternative medical explanatory concept (Fig. 2). This can be interpreted as a fatalistic view on mental health issues.

##### 4.2.2.4 Mental health understanding D: "biomedical (lifestyle focused)"

In this cluster, twenty-seven respondents (15,1%) demonstrated a high degree of confidence in the biomedical concept of “Lifestyle/behaviour/perception”. In contrast to physical health understanding type D, here the aforementioned concept is the only one where strong confidence is expressed in This conceptualisation exerts significant pressure on individuals grappling with mental health challenges, as it instils a sense of personal responsibility for their circumstances. Moderate confidence is expressed solely to other biomedical explanatory concepts (Fig. 2).

##### 4.2.2.5 Mental health understanding E: "absence of explanations"

The forty-five respondents (25,1%) of this cluster do not accept any explanatory concept for mental diseases. This prompts the hypothesis that the subjects question the very existence of mental health problems. It is hypothesised that at least a proportion of these respondents may dismiss mental health concerns as imagined issues (Fig. 2).

### 4.3 Further analyses and comparison of mental and physical health understandings

Exploratory bivariate analyses did not indicate significant associations between cluster membership and gender, educational level or age for either physical or mental health explanations.

An analysis of the distribution of participants across the different clusters of health understandings reveals a clear dominance of biomedical explanatory concepts among the respondents (Fig. 1, Fig. 2). Specifically, 81.6% of participants reported at least one formative biomedical explanatory concept regarding physical health (types B, C, D, and E), while 39.7% did so for mental health (types C and D). In contrast, supernatural and alternative medical explanatory concepts (at least) co-shaped 28.5% of physical and 35.2% of respondents’ mental health understandings (types A and B) (Fig. 1, Fig. 2).

A comparison of the total number of formative concepts shaping physical versus mental health understandings reveals a greater diversity in the former (Fig. 1, Fig. 2). Though the current data do not offer a definitive explanation, one possible interpretation is that stigma and feelings of shame may reduce im/migrants’ engagement with mental health topics, thereby limiting the development of corresponding formative explanatory concepts.

### 4.4 Respondents’ medical diversity

Medical diversity, understood as the participants’ borrowing of ideas, practices and styles from different medical ontologies becomes particularly evident through three key aspects: (1) an individual holds diverging understandings between physical and mental health; (2) an individual combines health understandings from different formative explanatory concepts rooted in disparate ontological perspectives; and (3) an individual holds a wide range of additional, merely 'accepted' explanatory concepts.

Contrary to our expectations, im/migrants’ understandings of physical and mental health in many cases varied significantly. A comparison of the participants’ understandings regarding physical and mental health shows that only understandings A, 'Supernatural powers', and B, 'Eclectic explanations', appear in both domains. All other clusters are distinct from one another, albeit to varying degrees. Consequently, the majority of participants (80.5%) holds divergent understandings of physical and mental health. It is important to note that the understandings of individual participants regarding physical and mental health do, at times, vary considerably.

**Fig. 3:**
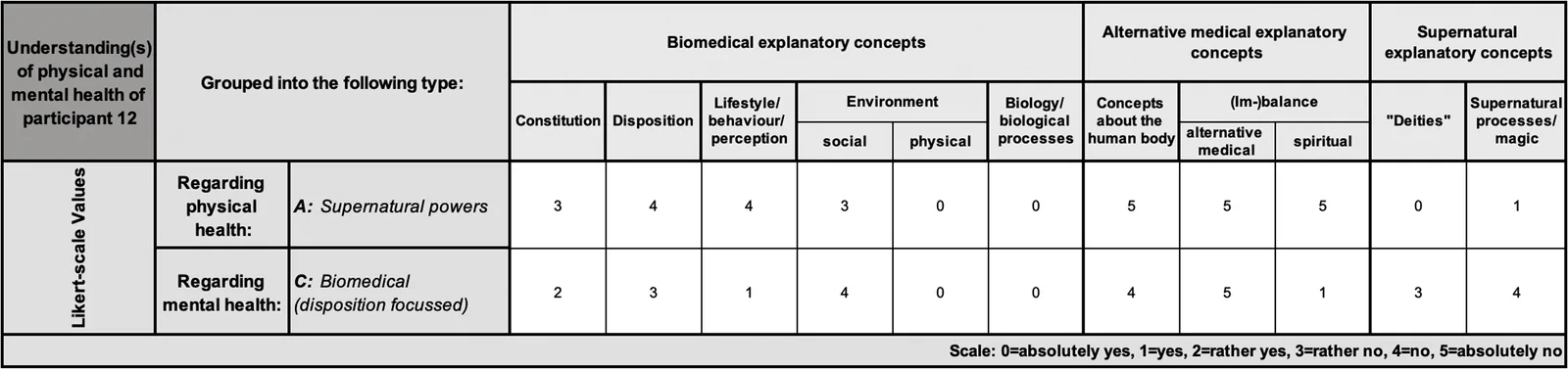
Understanding(s) of physical and mental health of participant 12.

The responses exhibited by participant number 12 are demonstrative of this phenomenon, as illustrated in Fig. 3. With regard to physical health, the participant employs a range of biomedical and supernatural explanatory concepts, while eschewing alternative medical concepts. Conversely, in relation to mental health, the participant draws upon biomedical and alternative medical concepts, while rejecting supernatural concepts.

The number of participants exhibiting the same understanding for both, physical and mental health, is relatively small (19.5%). This observation underscores a pronounced degree of medical diversity.

Considering the specific types of understandings identified, the medical diversity of im/migrants in Bonn-Tannenbusch and Cologne-Mülheim becomes even more apparent. Some respondents integrate multiple disparate formative health concepts, resulting in highly diverse health understandings. Type B of both typologies is particularly illustrative of this phenomenon, as it encompasses formative explanatory concepts from two categories of explanatory concepts.

Beyond this, all identified types encompass a range of 'accepted' explanatory concepts that, at least situationally, exert an influence on an individual's overall explanation of health and disease – and consequently influences the choice of health practices (Fig. 1, Fig. 2). When these accepted concepts are considered alongside the formative explanatory concepts, it becomes evident that they contribute to even more individualised health understandings.

Consequently, this study’s findings emphasise that medical diversity is not an exceptional occurrence among the study participants, but rather, a fundamental and pervasive component of their health understandings, profoundly shaping their everyday health practices.

## 5 Discussion

Previous studies, including those by Eisenbruch (1990); Foster (1976), and Murdock et al. (1980), have sought to categorise the wide range of understandings and theories of health that are prevalent in different societies. Others chose to categorise either largely biomedical or non-biomedical theories/beliefs and explanatory concepts for health (Erickson, 2008; Faltermaier et al., 1998). A preliminary typology of health understandings of im/migrants in Bonn-Tannenbusch and Cologne-Mülheim, has already been developed based on qualitative data (Becker et al., 2026). However, due to the methodological approach adopted, it was advised against the generalisation of these findings. The present study builds on this foundation using a quantitative methodology allowing for more generalisable results, despite the limitations arising from sampling and sample size. Inductively we developed more robust typologies of im/migrants’ understandings of physical and mental health in the same two neighbourhoods. The findings of the present study advance the earlier study’s findings in two aspects: a) the data analysis led to the identification of novel health understandings and b) it distinguishes clearly between understandings of physical and mental health. The latter allows for an even clearer identification of the respondents’ inherent medical diversity. Yet, the earlier study’s results are not obsolete, as they provide deeper insights into the respondents’ ontologies of health.

The types inductively constructed here are composed of explanatory concepts previously recognised as central to im/migrants’ health understandings. Consistent with earlier findings (Becker et al., 2026), these can be grouped into three categories: biomedical, alternative medical, and supernatural.

Biomedical explanatory concepts, which are particularly evident in types B, C, D, and E for physical health and in types C and D for mental health, have been mentioned in earlier works on health understandings of im/migrants in Germany. These include, for example, psychological dispositions (Franz et al., 2007; Shu et al., 2022) and social determinants like family relationships and work stressors (Franz et al., 2007; Grupp et al., 2021; Lujic, 2008; Shu et al., 2022; van der Meer et al., 2023; Yilmaz-Aslan et al., 2014), lifestyle, e.g., diet and exercise (van der Meer et al., 2023; Yilmaz-Aslan et al., 2014), emotional experiences, e.g., stress, loneliness (Grupp et al., 2018; Kikhia et al., 2021), physical environmental conditions (Lujic, 2008; Tezcan, 2018), and biological causes, e.g., genetic or microbial (Franz et al., 2007; Lujic, 2008; Shu et al., 2022; van der Meer et al., 2023).

Although less frequently addressed, previous research has also identified alternative medical (Dilger and Schnepf, 2020; Kotte, 2010) and supernatural explanatory concepts for health of im/migrants in Germany (Franz et al., 2007; Grupp et al., 2018; Lujic, 2008; van der Meer et al., 2023; Yilmaz-Aslan et al., 2014), which are inherent to types A and B of the typologies.

The mixed-methods research approach allowed for an advanced distinction between participants' understandings of physical and mental health. The findings of the present cross-sectional study were largely consistent with the results of the earlier qualitative study (Becker et al., 2026). While the types of the two typologies proposed here do not match precisely with those of our preceding work, we were able to confirm all of the previously identified types of health understandings being held by numerous or at least by selected participants. However, when compared to the results of other qualitative studies, the findings of this cross-sectional study suggest that biomedical explanatory concepts have a greater influence on the health understanding of im/migrants in Bonn-Tannenbusch and Cologne-Mülheim than was previously assumed. Concurrently, the influence of supernatural and alternative medical explanatory concepts on the health understanding of im/migrants in both neighbourhoods appears to be lower than expected.

The presence of multiple explanatory concepts within each of the identified types of health understandings, as well as the number of disparate types identified in the present work, highlight that "medical diversity" (Parkin, 2013, 125) is the norm and not an exception among im/migrants in Bonn-Tannenbusch and Cologne-Mülheim. Other scholars have described similar individual configurations of diverse health understandings and health practices of im/migrants using alternative terminologies such as "medical pluralism" or no specific terminology (Gilbert et al., 2019; Main, 2016; Samerski, 2019).

Ultimately, this study demonstrates the complex and multidimensional nature of im/migrants’ health understandings. It offers two comprehensive typologies through the explicit differentiation between physical and mental health. The identification of novel understandings underscores the necessity for additional empirical investigation.

## 6 Strengths and limitations

A key strength of this study lies in its methodological design. Compared to preceding qualitative work (Authors, 2026), the findings presented here are based on a larger quantitative survey and therefore allow for a more systematic assessment of patterns in health understandings among im/migrants in the two neighbourhoods. However, given the limitations arising from the sampling strategy, caution needs to be exerted regarding the generalisability of findings for the im/migrant population of the two neighbourhoods let alone the im/migrant population in Germany.

Another strength lies is the development of a data collection tool that can – after further application and refinement – be included in the growing repository of standardised instruments for health surveys. It proposes a novel set of variables, which allows to examine the various (in parts culturally influenced) explanatory concepts of health, the understandings of health composed from those concepts and the health practices, which are informed by those understandings and inherent concepts, among people with and without an im/migrant background. The conducted reliability analyses provide initial evidence of internal consistency for the theoretically derived multi-item dimensions. However, further applications and psychometric testing are required before the instrument can be considered standardised.

This study partly corroborated and further differentiated previously identified types of health understandings (Becker et al., 2026), resulting in two comprehensive typologies. In comparison with the qualitative typology, which in concerned with capturing fine-grained and, in some parts, rare configurations of health understandings (Becker et al., 2026), the cluster analyses identify broader empirically stable response patterns in the survey data. In particular, the separate analysis of understandings related to (1) physical and (2) mental health proved insightful, allowing for a more nuanced interpretation of the complexity and variability within im/migrants’ health understandings.

The present study is subject to several limitations. First, while the data collection tool was professionally translated into seven languages, this process was only completed after conducting the pretest using the German version and its English translation. Due to time constraints, pretests for the other language versions could not be carried out, which may have affected the comprehensibility and cultural appropriateness of the translated questionnaires.

Second, the necessary non-probabilistic element in the sampling limits the results generalisability. This is primarily due to the limited availability of comparative data on the im/migrant populations in the neighbourhoods of Bonn-Tannenbusch and Cologne-Mülheim. Moreover, existing data are often based on differing definitions of im/migration background, which deviate from the definition employed in this study, namely, individuals who have either im/migrated themselves or have at least one parent or grandparent who im/migrated to Germany. These inconsistencies in definition limit the comparability of the data and thus the ability to make a robust assessment of the sample’s representativeness.

Additionally, it is likely that not all subgroups within the neighbourhoods were equally reached during participant recruitment. For instance, only ten participants completed the survey in a language other than German, and individuals with higher educational attainment were overrepresented, as 62% of all participating im/migrants reported having a general and subject-specific entrance qualification for universities or a university or technical college degree as their highest educational qualification (Table 2).

This study proposes two typologies of im/migrants’ understandings of physical and mental health that both build on and partially diverges from our previous work (Becker et al., 2026). While the applied standardised methodology allowed for the identification of broader response patterns, further qualitative investigation is needed to better understand the rare and more case-specific health understandings of im/migrants that are less visible in the cluster-analyses.

Further, from the presented findings, we cannot conclude whether the identified types of health understandings and inherent explanatory concepts are to be understood as im/migrant-specific or whether they may also reflect the understandings of residents without an im/migration background. Jäckle & Timmis (2024), Dilger & Schnepf (2020) or Faltermaier et al. (1998), for example, recognised diverse health understandings without reference to an im/migration background. To answer this question was beyond the scope of this paper.

Future research should seek to (1) specify whether the identified types of health understandings are to be understood as migrant-specific, and (2) elucidate the manner in which im/migrants' health understandings are influenced by their membership within multiple (trans-)local social groups (Schatzki's, 1996, 168). As demonstrated in preceding studies, social groups have been shown to exert strong influence on the physical and mental health understandings and underlying explanatory concepts of im/migrants (e.g., Becker et al., 2026; Grupp et al., 2021; Shu et al., 2022; van der Meer et al., 2023).

## 7 Conclusion

In this study, we developed two comprehensive, empirically grounded typologies of im/migrants’ understandings of physical and mental health. In doing so we confirm and deepen prior insights regarding the complexity and diversity of im/migrants’ health understandings (Becker et al., 2026). It identifies five distinct (sub-)types of health understandings. These are influenced by biomedical, alternative medical, and supernatural explanatory concepts, which shape im/migrants' health practices. In addition to the aforementioned typology, five physical health understandings were identified: 'Supernatural powers', 'Eclectic explanations', 'Biological processes', Exposure and behaviour', 'Biomedical explanations'. In the context of mental health understanding, cluster analysis yielded the following five types of health understandings: 'Supernatural powers', 'Eclectic explanations', Biomedical (disposition focused)', Biomedical (lifestyle focused)', 'Absence of explanations'.

Following Schatzki’s practice-theory approach (1996, 2002), these types of health understandings, can be conceptualised as “general understandings” (2002, 77). They constitute an important precondition for the development of health practices and thus shape health care utilisation.

A key finding of this study is the high degree of medical diversity among im/migrants. This is evident not only in the broad range of formative and accepted explanatory concepts, which together form their identified disparate health understandings, but also in the observation that almost 80% of the surveyed im/migrants do not adhere to a single, coherent understanding in the physical and the mental health domain. Instead, individuals frequently draw on, in parts, entirely different explanatory concepts. This underlines the situational and contextual character of health understandings.

Biomedical concepts, such as disposition, biological processes, environmental factors, and behavioural aspects, dominate the health understandings of the majority of respondents, especially regarding physical health. Nonetheless, supernatural and alternative medical concepts play an important role, particularly in the domain of mental health, where stigma and social silence may limit engagement with biomedical explanatory concepts and create space for other frameworks.

By distinctly analysing physical and mental health understandings, this study not only enriches the conceptualisation of im/migrants’ health understandings but also demonstrates that medical diversity is a pervasive and constitutive feature of im/migrant health understandings.

Both typologies introduced here can serve as analytical tools for future research and the design of interventions, especially in public health communication, health education, and the design of culturally sensitive care models. Recognizing and responding to the diversity of im/migrants' health understandings is essential to ensure that health services are inclusive, respectful, and effective.

## Ethical considerations

The Ethics Committee of the Medical Faculty of Cologne approved the project in general on August 25, 2022 (application number: 22–1176), and the quantitative methodology utilised in its second research phase on June 11, 2024 (application number: 22–1176_3).

## Funding statement

This work was supported by the 10.13039/501100001736German Research Foundation (DFG) [grant number: 505387561].

## Ethics declaration

Written informed consent to take part in the study and to publish the article has been obtained from all participants or their legal representatives. The privacy rights of participants have been observed.

This study was performed in compliance with relevant laws, regulatory frameworks and guidelines where the research took place. This study was approved by the Ethics Committee of the Medical Faculty of Cologne. (Approval No 22–1176_3)

## CRediT authorship contribution statement

**Kevin Becker:** Writing – review & editing, Writing – original draft, Visualization, Methodology, Investigation, Formal analysis, Conceptualization. **Frauke Kraas:** Writing – review & editing, Supervision, Resources, Project administration, Funding acquisition. **Jonas Birke:** Writing – review & editing, Formal analysis. **Carsten Butsch:** Writing – review & editing, Supervision, Project administration, Funding acquisition, Formal analysis, Conceptualization.

## Declaration of competing interest

The authors declare the following financial interests/personal relationships which may be considered as potential competing interests:

Kevin Becker reports financial support was provided by German Research Foundation. Frauke Kraas reports financial support was provided by German Research Foundation. Jonas Birke reports no financial support was provided for this research. Carsten Butsch reports financial support was provided by German Research Foundation.

## Appendix Supplementary materials

Image, application 1

Image, application 2

Image, application 3
