# Supplementary material for: Medical diversity of migrants’ physical and mental health understandings
Source: J Migr Health. 2026 Aug 22;14:100438. doi: 10.1016/j.jmh.2026.100438 (PMC13543081; doi:10.1016/j.jmh.2026.100438)
Supplement: Supplementary file 1 [file mmc1.pdf]

Health understandings and health practices in Bonn-Tannenbusch

Blinded for review

Warum werde ich krank und was macht mich gesund?

Neden hastalanıyorum ve beni ne iyileştirecek?

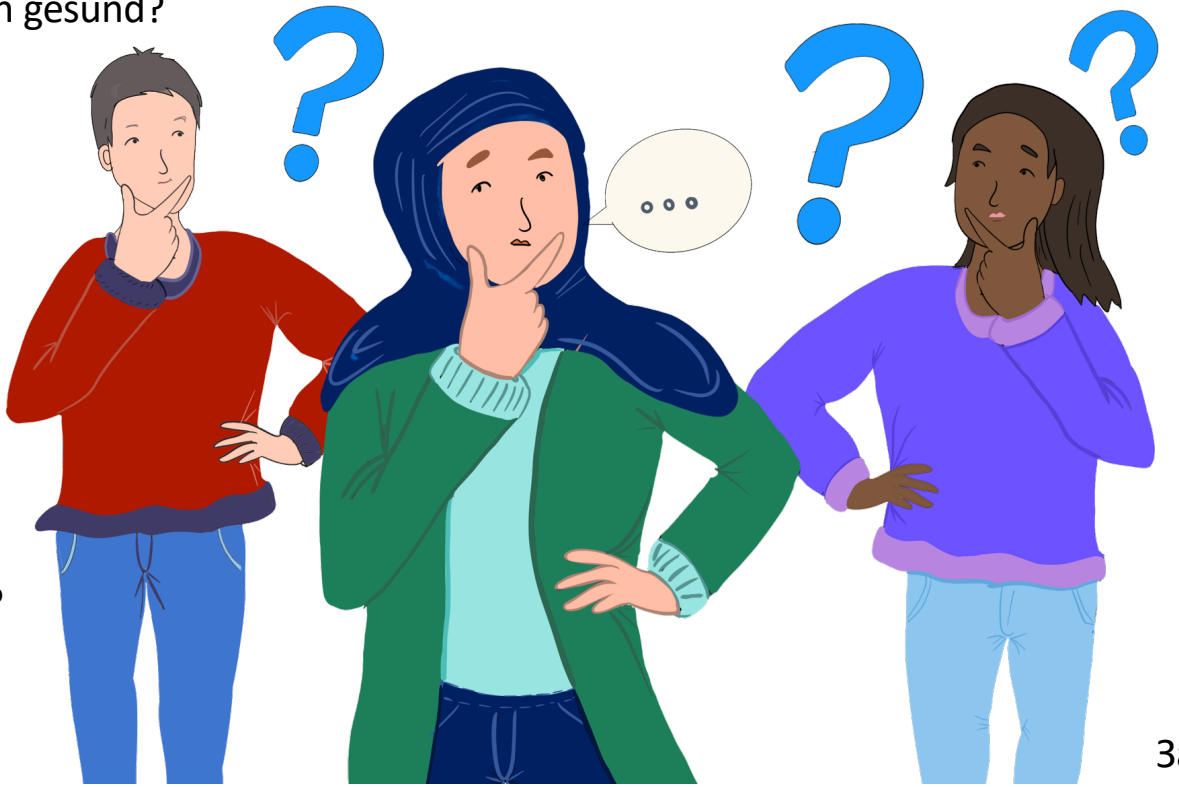

Why do I get ill and how do I regain my health?

لماذا أصاب بالمرض وما الذي يجعلني أتعافى؟

De ce mă îmbolnăvesc și ce mă face însănătoșește?

Почему я болею и что делает меня здоровым?

Защо се разболявам и какво ме лекува?

Dlaczego zapadam na choroby i co sprawia, że zdrowieję?

جرا بیمار می‌شوم و از چه راهی سلامت خود را بازمی‌یابم؟

Blinded for review

Blinded for review

Wir laden Sie ein, an einer Befragung zu unterschiedlichen Vorstellungen von Gesundheit, Krankheit und Heilung von Bewohner*innen in Bonn-Tannenbusch teilzunehmen. Den Fragebogen erreichen Sie über den QR-Code oder nutzen Sie den Link.

ندعوك للمشاركة في استطلاع رأي حول التصورات المختلفة عن الصحة والمرض والشفاء بين السكان في بون-تانينبوش. يمكنك الوصول إلى الاستبيان عبر رمز الاستجابة السريعة (QR-Code) أو استخدام الرابط.

We invite you to participate in a survey about diverse perceptions of health, disease and healing among the residents of Bonn-Tannenbusch. You can access the questionnaire using the QR-code or the link.

Sizi Bonn-Tannenbusch sakinleri arasında sağlık, hastalık ve iyileşmeye ilişkin farklı algılar üzerine bir ankete katılmaya davet ediyoruz. Ankete QR kodu üzerinden veya bağlantıyı kullanarak erişebilirsiniz.

Blinded for review

Zapraszamy do udziału w ankiecie na temat różnych wyobrażeń na temat zdrowia, choroby i procesu zdrowienia, którą przeprowadzamy wśród mieszkańców dzielnicy Bonn-Tannenbusch. Ankieta jest dostępna poprzez kod QR albo pod podanym linkiem.

از شما برای شرکت در نظرسنجیِ برداشت و تلقی شما از سلامت، بیماری و درمان در بین ساکنین مولهایم-کلن دعوت می‌کنیم. با اسکن کیوآرکد یا مراجعه به این لینک می‌توانید در نظرسنجی شرکت نمایید.

Каним Ви да участвате в анкета относно различни представи за здраве, заболяване и лечение на жителите(ките) на Бон-Таненбуш. За достъп до анкетния лист използвайте QR кода или линка.

Приглашаем вас принять участие в опросе о различных представлениях жителей Бонн-Танненбуш о здоровье, заболеваниях и исцелении. Вы можете получить доступ к анкете с помощью QR-кода или воспользоваться ссылкой.

Blinded for review

Vă invităm să participați la un sondaj privind diferitele percepții ale sănătății, bolii și vindecării în rândul locuitorilor din Bonn-Tannenbusch. Puteți ajunge la chestionar prin intermediul codului QR sau puteți utiliza linkul.

Blinded for review
