# Supplementary material for: Medical diversity of migrants’ physical and mental health understandings
Source: J Migr Health. 2026 Aug 22;14:100438. doi: 10.1016/j.jmh.2026.100438 (PMC13543081; doi:10.1016/j.jmh.2026.100438)
Supplement: Supplementary file 2 [file mmc2.pdf]

Health understandings and health practices in Cologne-Mülheim

Blinded for review

Warum werde ich krank und was macht mich gesund?

Neden hastalanıyorum ve beni ne iyileştirecek?

Why do I get ill and how do I regain my health?

لماذا أصاب بالمرض وما الذي يجعلني أتعافى؟

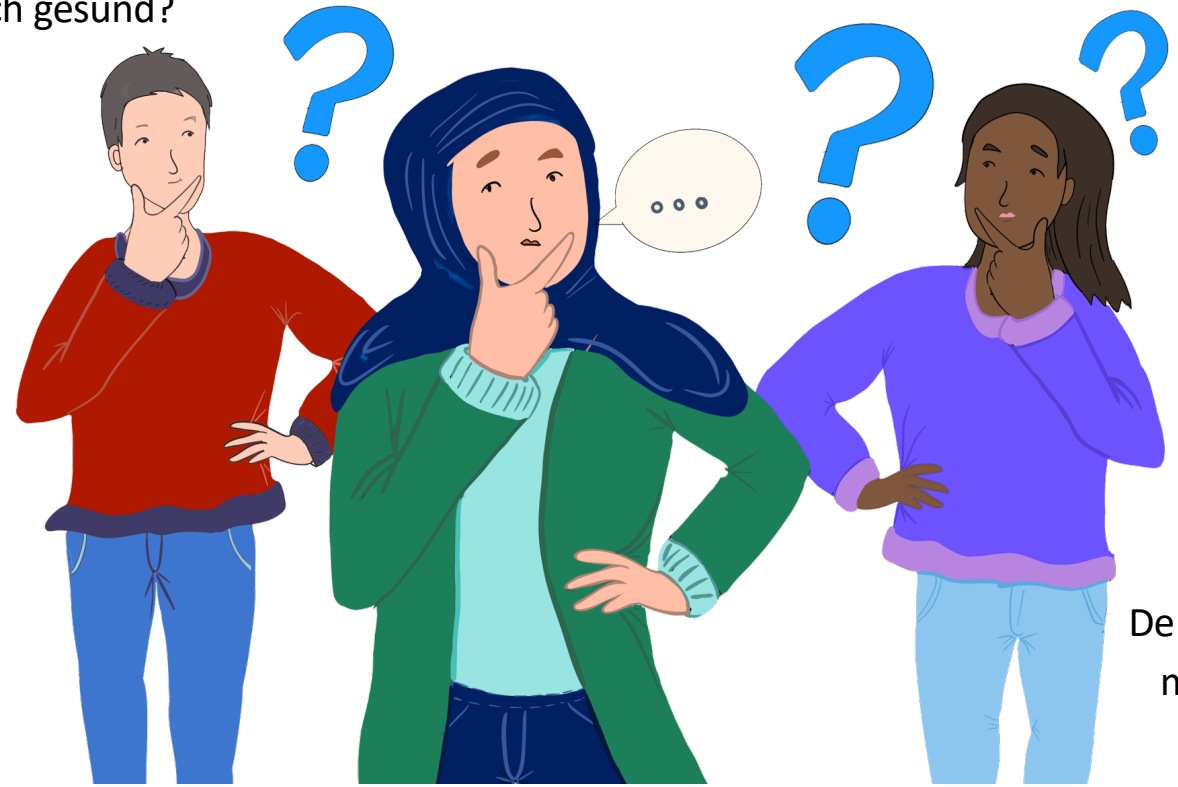

Почему я болею и что делает меня здоровым?

Dlaczego zapadam na choroby i co sprawia, że zdrowieję?

De ce mă îmbolnăvesc și ce mă face însănătoșește?

چرا بیمار می‌شوم و از چه راهی سلامت خود را بازمی‌یابم؟

Защо се разболявам и какво ме леку[redacted]

Blinded for review

Blinded for review

Wir laden Sie ein, an einer Befragung zu unterschiedlichen Vorstellungen von Gesundheit, Krankheit und Heilung von Bewohner*innen in Köln-Mülheim teilzunehmen. Den Fragebogen erreichen Sie über den QR-Code oder nutzen Sie den Link.

ندعوك للمشاركة في استطلاع رأي حول التصورات المختلفة عن الصحة والمرض والشفاء بين السكان في كولونيا مولهايم. يمكنك الوصول إلى الاستبيان عبر رمز الاستجابة السريعة (QR-Code) أو استخدام الرابط.

We invite you to participate in a survey about diverse perceptions of health, disease and healing among the residents of Cologne-Mülheim. You can access the questionnaire using the QR-code or the link.

Sizi Köln-Mülheim sakinleri arasında sağlık, hastalık ve iyileşmeye ilişkin farklı algılar üzerine bir ankete katılmaya davet ediyoruz. Ankete QR kodu üzerinden veya bağlantıyı kullanarak erişebilirsiniz.

Blinded for review

Zapraszamy do udziału w ankiecie na temat różnych wyobrażeń na temat zdrowia, choroby i procesu zdrowienia, którą przeprowadzamy wśród mieszkańców dzielnicy Köln-Mülheim. Ankieta jest dostępna poprzez kod QR albo pod podanym linkiem.

از شما برای شرکت در نظرسنجیِ برداشت و تلقی شما از سلامت، بیماری و درمان در بین مولهایم-کلن دعوت می‌کنیم. با اسکن کیوآرکد یا مراجعه به این لینک می‌توانید در نظرسنجی شرکت نمایید.

Каним Ви да участвате в анкета относно различни представи за здраве, заболяване и лечение на жителите(ките) на Кьолн-Мюлхайм. За достъп до анкетния лист използвайте QR кода или линка.

Приглашаем вас принять участие в опросе о различных представлениях жителей Кельн-Мюльхайма о здоровье, заболеваниях и исцелении. Вы можете получить доступ к анкете с помощью QR-кода или воспользоваться ссылкой.

Vă invităm să participați la un sondaj privind diferitele percepții ale sănătății, bolii și vindecării în rândul locuitorilor din Köln-Mülheim. Puteți ajunge la chestionar prin intermediul codului QR sau puteți utiliza

Blinded for review

Blinded for review
