# Supplementary material for: Medical diversity of migrants’ physical and mental health understandings
Source: J Migr Health. 2026 Aug 22;14:100438. doi: 10.1016/j.jmh.2026.100438 (PMC13543081; doi:10.1016/j.jmh.2026.100438)
Supplement: Supplementary file 3 [file mmc3.pdf]

Survey: Health in diverse neighborhoods

Dear Ladies, dear Gentlemen,
thank you for your interest in our study about health in diverse neighborhoods. Your participation in the survey is very valuable to us.
We are currently in research phase II. We are investigating the diverse health perceptions and treatment methods in Bonn-Tannenbusch and Cologne-Mülheim. One method of data collection is an online survey of people who live in either Cologne-Mülheim or Bonn-Tannenbusch.
Further information on our study can be found on our website or in the privacy policy below.
Thank you for your participation.

Please read the statements of the following people carefully. Which person would you agree with the most? Please choose only one of the following:

	Person 1: God/Allah has an influence on my health. Health is a gift from God/Allah and God/Allah protects me from disease. Diseases can be a test from God/Allah or God/Allah wants to punish me with it. Diseases can also be caused by jinns, demons and other evil supernatural beings or by shamans, sorcerers and wizards.
	Person 2: Like person 1, I believe that God and black magic (jinns, demons, shamanism and sorcery) influence my health. However, I believe that my behavior can also affect my health, because if I smoke, drink alcohol or drive too fast, for example, my health can be affected.
	Person 3: I am healthy when my body and soul are in balance. This balance exists above all when I have a good relationship with God/Allah and my faith. I maintain this spiritual balance, and therefore my health, when I live in harmony with God/Allah. Diseases are the result of a lack of spiritual balance, which persists until my body and soul are in balance again.
	Person 4: I am healthy when there is balance in my body or when my body and my environment are in balance. Here I mean, for example, a balanced presence of bodily fluids or a balanced presence of energies in my body. If I have a cold, for example, there is too much "cold" in my body, which is why I eat hot or spicy food or drink or go to the sauna/hamam.
	Person 5: Diseases are usually caused by infections with bacteria or viruses. There are also diseases that are inherited. I also believe that a strong body and a strong mind are less susceptible to diseases. Likewise, my behavior (e.g. smoking, taking alcohol, driving fast) can affect my health. My environment can also have positive and negative effects on my health (good or bad social relationships, fresh air or car exhaust fumes).

Understanding of health and disease

In the following, explanations of exclusively PHYSICAL DISEASES are asked for.
Please choose only one of the following:

	Understood

You have a disease and suffer from strong headaches. How do you explain the origin of the disease?

	Do you agree with the statement? Your answer must be between 0 (absolutely yes) and 5 (absolutely no)						Compared to your childhood and youth, do you believe less, equally or more in this idea today? Your answer must be between 0 (less) and 3 (unsure)			
**"Diseases occur from a poor physical condition, which is inherited."**	Absolutely yes	yes	Rather yes	Rather no	No	Absolutely no	Less	Same	More	Unsure
**"Diseases occur by a poor mental condition, e.g. when you are stressed or sad."**	Absolutely yes	yes	Rather yes	Rather no	No	Absolutely no	Less	Same	More	Unsure
**"Diseases occur when I behave incorrectly (e.g. smoking, unhealthy diet, lack of exercise), everyone can influence their own health."**	Absolutely yes	yes	Rather yes	Rather no	No	Absolutely no	Less	Same	More	Unsure
**"Diseases occur when I argue with my family and friends. "**	Absolutely yes	yes	Rather yes	Rather no	No	Absolutely no	Less	Same	More	Unsure
**"Various things from the environment are causing diseases (e.g. car exhaust fumes, noise or environmental toxins)."**	Absolutely yes	yes	Rather yes	Rather no	No	Absolutely no	Less	Same	More	Unsure
**"Diseases are inherited or are transmitted by viruses or bacteria, for example."**	Absolutely yes	yes	Rather yes	Rather no	No	Absolutely no	Less	Same	More	Unsure
**"Diseases occur from impure and bad blood or when blockages interrupt the energy flow in my body."**	Absolutely yes	yes	Rather yes	Rather no	No	Absolutely no	Less	Same	More	Unsure
**"Diseases occur when my soul is out of balance."**	Absolutely yes	yes	Rather yes	Rather no	No	Absolutely no	Less	Same	More	Unsure
**"Diseases occur when there is an imbalance in my body (e.g. between "hot" and "cold" or between "yin" and "yang")."**	Absolutely yes	yes	Rather yes	Rather no	No	Absolutely no	Less	Same	More	Unsure
**"Diseases are the will of God."**	Absolutely yes	yes	Rather yes	Rather no	No	Absolutely no	Less	Same	More	Unsure
**"Evil Eye (Nazar), Jinns, demons or even fate can cause disease."**	Absolutely yes	yes	Rather yes	Rather no	No	Absolutely no	Less	Same	More	Unsure

In the following, explanations of exclusively NON-PHYSICAL DISEASES ("mental" diseases) are asked for. Please choose only one of the following:

	Understood

You have a non-physical disease and suffer from strong inner tension and restlessness, for example. How do you explain the origin of the desease?

	Do you agree with the statement? Your answer must be between 0 (absolutely yes) and 5 (absolutely no)						Compared to your childhood and youth, do you believe less, equally or more in this idea today? Your answer must be between 0 (less) and 3 (unsure)			
**"Non-physical diseases occur from a poor physical condition, which is inherited."**	Absolutely yes	yes	Rather yes	Rather no	No	Absolutely no	Less	Same	More	Unsure
**"Non-physical diseases occur by a poor mental condition, e.g. when you are stressed or sad."**	Absolutely yes	yes	Rather yes	Rather no	No	Absolutely no	Less	Same	More	Unsure
**"Non-physical diseases occur when I behave incorrectly (e.g. smoking, unhealthy diet, lack of exercise), everyone can influence their own health."**	Absolutely yes	yes	Rather yes	Rather no	No	Absolutely no	Less	Same	More	Unsure
**"Non-physical diseases occur when I argue with my family and friends."**	Absolutely yes	yes	Rather yes	Rather no	No	Absolutely no	Less	Same	More	Unsure
**"Various things from the environment make cause diseases and can cause non-physical diseases (e.g. car exhaust fumes, noise or environmental toxins)."**	Absolutely yes	yes	Rather yes	Rather no	No	Absolutely no	Less	Same	More	Unsure
**"Non-physical diseases are inherited or are transmitted by viruses or bacteria, for example."**	Absolutely yes	yes	Rather yes	Rather no	No	Absolutely no	Less	Same	More	Unsure
**"Non-physical diseases occur from impure and bad blood or when blockages interrupt the energy flow in my body."**	Absolutely yes	yes	Rather yes	Rather no	No	Absolutely no	Less	Same	More	Unsure
**"Non-physical diseases occur when my soul is out of balance."**	Absolutely yes	yes	Rather yes	Rather no	No	Absolutely no	Less	Same	More	Unsure
**"Non-physical diseases occur when there is an imbalance in my body (e.g. between "hot" and "cold" or between "yin" and "yang")."**	Absolutely yes	yes	Rather yes	Rather no	No	Absolutely no	Less	Same	More	Unsure
**"Non-physical diseases are the will of God."**	Absolutely yes	yes	Rather yes	Rather no	No	Absolutely no	Less	Same	More	Unsure
**"Evil Eye (Nazar), Jinns, demons or even fate can cause non-physical disease."**	Absolutely yes	yes	Rather yes	Rather no	No	Absolutely no	Less	Same	More	Unsure

Which statement applies most to you? Please choose only one of the following:

	My ideas have changed because there are other explanations in Germany that are more convincing.
	My ideas have changed because I have developed my own ideas as I have grown older. I have let go of ideas that my parents taught me.
	Contact with people from other societies of origins has led to my ideas changing.
	My ideas have changed because my religious beliefs have changed.
	I have abandoned previous ideas because they could no longer explain my suffering..

If you have learned new ideas about the causes of health and disease, through whom (or what) did this happen? Please choose all that apply:

	I have not learned any new ideas about the causes of health and disease.
	Partner, parents or other family members
	Friends with a migration background, own society of origin
	Friends with a migration background, other society of origin
	Friends without a migration background, from Germany
	Neighbours with a migration background, own society of origin
	Neighbours with a migration background, other society of origin
	Neighbours without a migration background, from Germany
	Doctor
	Specialist doctor *in
	(Natural) healer, homeopath (etc.)
	Traditional healer, shaman, witch (etc.)
	Imam, Hodja, Priest (etc.)
	Teacher or other employee of an educational institution
	Through books or magazines
	Via the Internet

Where do you talk to other people about why you get sick, get healthy or stay healthy? Please choose **all** that apply:

	There is no place where I discuss with other people why one gets sick, gets healthy or stays healthy.
	In Germany (with friends, in school, in parks etc.)
	In my society of origin, via telephone, WhatsApp, Skype/Zoom/FaceTime etc.
	In my home society of origin when I travel there.

Where do you talk to other people about why people get sick, get healthy or stay healthy?

	**No**	**In your district (Bonn-Tannenbusch/Cologne-Mülheim)**	**At your residence (Bonn or Cologne)**	**In another city**
**In private homes (of family or friends)**				
**In cafes, restaurants, tea houses etc.**				
**In facilities of migrant organisations**				
**In doctor's offices or other medical facilities**				
**In gyms and other sports facilities**				
**At school or other educational institutions**				

**On playgrounds or e.g. in parks**				
**n religious places (e.g. mosque, church, temple, synagogue, etc.)**				
**At the place of a healer**				
**Somewhere else**				

General information

In what year were you born? Please choose only one of the following:

	[Drop-down menu ranging from „1900“ to „after 20008“]

Which gender are you? Please choose only one of the following:

	Male
	Female
	Diverse

What is your highest level of education in Germany or your society of origin? Please choose only one of the following:

	No degree
	Secondary school certificate ("Hauptschulabschluss" or equivalent qualification)
	Intermediate school leaving certificate ("Mittlere Reife" or comparable qualification)
	University of Applied Sciences entrance qualification ("Fachhochschulreife" or equivalent qualification)
	General or subject-specific higher education entrance qualification (Abitur) (or comparable qualification)
	Degree from a technical college or university (or equivalent qualification)
	Completed vocational/professional training

Which society (e.g. Kurdish Turkish) do you and your parents feel you belong to most? Please choose only one of the following:

Self (Preselection)	Parent 1 (Preselection)	Parent 2 (Preselection)	
/			Parent non-existent
			Other
			Afghanistan
			Afghanistan (Pashtun)
			Afghanistan (Tajik)
			Afghanistan (Other)
			Algeria
			Algeria (Arabic)
			Algeria (Berber)
			Algeriia (Other)
			Bulgaria
			Germany
			Greece
			Iraq
			Iraq (Arabic)
			Irak (Kurdish)
			Irak (Other)
			Iran

			Iran (Persian)
			Iran (Kurdish)
			Iran (Other)
			Italy
			Marocco
			Marocco (Arabic)
			Marocco (Berber)
			Marocco (Other)
			Poland
			Romania
			Russia
			Somalia
			Syria
			Syria (Arabic)
			Syria (Alevi)
			Syria (Kurdish)
			Syria (Levantine)
			Syria (Other)
			Tunisia
			Turkey
			Turkey (Alevi)
			Türkei (Sunni)
			Türkei (Kurdish)
			Türkei (Other)
			Ukraine

Other society of origin?
Only answer this question if the following conditions are met:
Answer was 'Other' at question before.
Please write your answer here:

Self	Parent 1	Parent 2

Do you, your parents or grandparents have migration experience? Please choose only one of the following:

	No migration experience in the family
	Migrated in adulthood
	Migrated as a child
	One parent migrated, myself born in Germany
	Both parents migrated, myself born in Germany
	One or more grandparents migrated, parents and myself born in Germany

How many years have you been living in Germany?
Only answer this question if the following conditions are met: Answer was 'Migrated in adulthood' *or* 'Migrated as a child' at question before.
Please write your answer here:

How many years have you lived in Bonn-Tannenbusch/Cologne-Mülheim? Please write your answer here:

With People from which society of origin do you talk on a daily basis?

	Mostly from my society of origin
	Often with my society of origin
	Often with other society of origin
	Mostly with other society of origin

Do you feel at home here or do you feel more at home in the region of your society of origin? Only answer this question if the following conditions are met: Answer was NOT 'No migration experience in the family' at question 'Do you, your parents or grandparents have migration experience?)
Please choose **only one** of the following:

	I feel equally at home in Germany and in my society of origin
	I feel completely at home in my society of origin
	I feel rather at home in my society of origin
	I feel rather at home in Germany
	I feel completely at home in Germany

Can you communicate reliably in German?

	0- Absolutely reliable
	1- Reliable
	2- Rather reliable
	3- Rather unreliable
	4- Unreliable
	5- I do not speak German

Are you a religious person?

	0- Absolutely religious
	1- Very religious
	2- Rather religious
	3- Religious
	4- Not religious
	5- Absolutely not religious

To which of the following religions, beliefs or worldviews do you belong (most strongly)?
Please choose **only one** of the following:

	Christianity (Roman Catholic)
	Christianity (Protestant)

	Christianity (Orthodox)
	Islam (Sunni)
	Islam (Shiite)
	Islam (Alevi)
	Judaism
	Buddhism
	Hinduism
	Another religion
	No religious affiliation

How high is your test in the pharmaceutical industry? (medicine industry)?

	0- Very high
	1- High
	2- Rather high
	3- Rather low
	4- Low
	5- Very low

Treatment methods

In the following, treatment methods of exclusively PHYSICAL DISEASES are asked for. Please choose only one of the following:

	Understood

You have a disease and suffer from strong headaches. What do you do to get healthy and stay healthy?

the disease?

	Do you agree with the statement? Your answer must be between 0 (absolutely yes) and 5 (absolutely no)						Compared to your childhood and youth, do you believe less, equally or more in this idea today? Your answer must be between 0 (less) and 3 (unsure)			
**"If I have strong headaches, I spend time in nature (in the fresh air)."**	Absolutely yes	yes	Rather yes	Rather no	No	Absolutely no	Less	Same	More	Unsure
**"If I have strong headaches, I do a lot of sport (e.g. jogging, gym or Pilates) and eat a healthy diet."**	Absolutely yes	yes	Rather yes	Rather no	No	Absolutely no	Less	Same	More	Unsure
**"If I have strong headaches, I avoid stressful situations (e.g. at work)."**	Absolutely yes	yes	Rather yes	Rather no	No	Absolutely no	Less	Same	More	Unsure
**"If I have strong headaches, I look for social contact and social support (e.g. in my family)."**	Absolutely yes	yes	Rather yes	Rather no	No	Absolutely no	Less	Same	More	Unsure
**"If I have strong headaches, I take medication or carry out therapies."**	Absolutely yes	yes	Rather yes	Rather no	No	Absolutely no	Less	Same	More	Unsure
**"If I have strong headaches, I try to bring my body into balance, e.g. by dieting or exercising."**	Absolutely yes	yes	Rather yes	Rather no	No	Absolutely no	Less	Same	More	Unsure
**"For strong headaches, I take natural substances (e.g. medicinal plants) or alternative medicine (e.g. homeopathic remedies)."**	Absolutely yes	yes	Rather yes	Rather no	No	Absolutely no	Less	Same	More	Unsure
**"If I have strong headaches, I use "cupping" (hacamat) to remove bad and impure blood from the body or also carry out treatments to release blockages that hinder the energy flow."**	Absolutely yes	yes	Rather yes	Rather no	No	Absolutely no	Less	Same	More	Unsure
**"If I have strong headaches, I use rituals or wear pieces of jewelry and amulets (e.g. nazar amulet)."**	Absolutely yes	yes	Rather yes	Rather no	No	Absolutely no	Less	Same	More	Unsure
**"If I have strong headaches, I pray or read holy writings."**	Absolutely yes	yes	Rather yes	Rather no	No	Absolutely no	Less	Same	More	Unsure

**“If I have strong headaches, I consult traditional healers, sorcerers, witches or shamans and do what they tell me."**	Absolutely yes	yes	Rather yes	Rather no	No	Absolutely no	Less	Same	More	Unsure
**"If I have strong headaches, I use traditional rituals that heal illnesses caused by spirits, demons and jinns."**	Absolutely yes	yes	Rather yes	Rather no	No	Absolutely no	Less	Same	More	Unsure

In the following, threatment methods of exclusively NON-PHYSICAL DISEASES ("mental" diseases) are asked for.

	_Understood

You have a disease and suffer from strong inner tension and restlessness. What do you do to get healthy and stay health?

	**Do you agree with the statement? Your answer must be between 0 (absolutely yes) and 5 (absolutely no)**						**Compared to your childhood and youth, do you believe less, equally or more in this idea today? Your answer must be between 0 (less) and 3 (unsure)**			
**"If I suffer from strong inner tension and restlessness, I spend time in nature (in the fresh air)."**	Absolutely yes	yes	Rather yes	Rather no	No	Absolutely no	Less	Same	More	Unsure
**"If I suffer from strong inner tension and restlessness, I do a lot of sport (e.g. jogging, gym or Pilates) and eat a healthy diet"**	Absolutely yes	yes	Rather yes	Rather no	No	Absolutely no	Less	Same	More	Unsure
**"If I suffer from strong inner tension and restlessness, I avoid stressful situations (e.g. at work)."**	Absolutely yes	yes	Rather yes	Rather no	No	Absolutely no	Less	Same	More	Unsure
**"If I suffer from strong inner tension and restlessness, I look for social contact and social support (e.g. in my family)."**	Absolutely yes	yes	Rather yes	Rather no	No	Absolutely no	Less	Same	More	Unsure
**"If I suffer from strong inner tension and restlessness, I take medication or carry out therapies."**	Absolutely yes	yes	Rather yes	Rather no	No	Absolutely no	Less	Same	More	Unsure
**"If I suffer from strong inner tension and restlessness, I try to bring my body into balance, e.g. by dieting or exercising."**	Absolutely yes	yes	Rather yes	Rather no	No	Absolutely no	Less	Same	More	Unsure
**"If I suffer from strong inner tension and restlessness, I take natural substances (e.g. medicinal plants) or alternative medicine (e.g. homeopathic remedies)."**	Absolutely yes	yes	Rather yes	Rather no	No	Absolutely no	Less	Same	More	Unsure
**"If I suffer from strong inner tension and restlessness, I use "cupping" (hacamat) to remove bad and impure blood from the body or also carry out treatments to release blockages that hinder the energy flow."**	Absolutely yes	yes	Rather yes	Rather no	No	Absolutely no	Less	Same	More	Unsure

**"If I suffer from strong inner tension and restlessness, I use rituals or wear pieces of jewelry and amulets (e.g. nazar amulet)."**	Absolutely yes	yes	Rather yes	Rather no	No	Absolutely no	Less	Same	More	Unsure
**"If I suffer from strong inner tension and restlessness, I pray or read holy writings."**	Absolutely yes	yes	Rather yes	Rather no	No	Absolutely no	Less	Same	More	Unsure
**"If I suffer from strong inner tension and restlessness, I consult traditional healers, sorcerers, witches or shamans and do what they tell me."**	Absolutely yes	yes	Rather yes	Rather no	No	Absolutely no	Less	Same	More	Unsure
**"If I suffer from strong inner tension and restlessness, I use traditional rituals that heal illnesses caused by spirits, demons and jinns."**	Absolutely yes	yes	Rather yes	Rather no	No	Absolutely no	Less	Same	More	Unsure

Why have the treatment methods you use today changed? Which statement is most accurate? Please answer this question only if at least one of the questions above was answered with "more" or "less." Please choose **only one** of the following:

	I have learned about new treatment methods that I think are more promising.
	I have learned about new treatment methods that I consider to be safer (fewer side effects).
	The treatment methods used previously did not work quickly enough or did not lead to a cure.
	As my understanding of health has changed, the treatment methods I use have also changed.

Have you ever tried multiple treatments for a disease at the same time? Please choose only one of the following:

	Yes
	No

Why did you use multiple treatment methods at the same time for a disease? Only answer this question if the following conditions are met: Answer was 'Yes' at question 'Have you ever tried multiple treatments for a disease at the same time? " Please choose **all** that apply:

	If I use many treatment methods, I will recover faster.
	I don't not know which treatment method is best, so I am trying out different methods.
	I use the methods recommended by friends and family, but at the same time I also trust the methods of healthcare providers.
	I use the treatment methods that I used as a child. At the same time, however, I also rely on methods that I have recently learned about.

Where do you mainly use health services? Please choose all that apply:

	I do not use any health services
	In Germany (with friends, in school, in parks etc.)
	n my society of origin, via telephone, WhatsApp, Skype/Zoom/FaceTime etc.
	In my society of origin when I travel there

Where do you mainly use health services?

	**No**	**In your district (Bonn-Tannenbusch/Cologne-Mülheim)**	**At your residence Bonn/Cologne)**	**In another city**
**In private homes (of family or friends)**				
**In facilities of migrant organisations**				

**In doctor's offices or other medical facilities**				
**In gyms and other sports facilities**				
**In religious places (e.g. mosque, church, temple, synagogue, etc.)**				
**At the place of a healer**				
**Somewhere else**				

If you have learned about new treatment methods, who (or what) introduced you to them? Please choose all that apply:

	I have not learned any new treatment methods
	Partner, parents or other family members
	Friends with a migration background, own society of origin
	Friends with a migration background, other society of origin
	Friends without a migration background, from Germany
	Neighbours with a migration background, own society of origin
	Neighbours with a migration background, other society of origin
	Neighbours with a migration background, other society of origin
	Doctor
	Specialist doctor
	(Natural) healer, homeopath (etc.)
	Traditional healer, shaman, witch (etc.)
	Imam, Hodja, Priest (etc.)
	Teacher or other employee of an educational institution
	Through books or magazines
	Via the Internet

Thank you for your participation!

A project newsletter will inform you about changes in the study process and the progress of the study. If you would like to receive the newsletter, please enter your email address here.
(Your contact details will be stored separately from the rest of the data so that your information remains anonymous.)

Please write your answer here:

If you would like to participate in future research phases of the study, please enter your telephone number here.
(Your contact details will be stored separately from the rest of the data so that your information remains anonymous.)

Please write your answer here:

